# Playing vs. Nonplaying Aerobic Training in Tennis: Physiological and Performance Outcomes

**DOI:** 10.1371/journal.pone.0122718

**Published:** 2015-03-27

**Authors:** Vincent Pialoux, Cyril Genevois, Arnaud Capoen, Scott C. Forbes, Jordan Thomas, Isabelle Rogowski

**Affiliations:** 1 Université de Lyon, Université Lyon 1, Centre de Recherche et d'Innovation sur le Sport—EA 647; UFRSTAPS; Villeurbanne, France; 2 Institut Universitaire de France, Paris, France; 3 Ligue de l’Essonne de Tennis, Ste Geneviève des Bois, France; 4 Human Kinetics, Okanagan College, Penticton, British Columbia, Canada; University of the Balearic Islands, SPAIN

## Abstract

This study compared the effects of playing and nonplaying high intensity intermittent training (HIIT) on physiological demands and tennis stroke performance in young tennis players. Eleven competitive male players (13.4 ± 1.3 years) completed both a playing and nonplaying HIIT session of equal distance, in random order. During each HIIT session, heart rate (HR), blood lactate, and ratings of perceived exertion (RPE) were monitored. Before and after each HIIT session, the velocity and accuracy of the serve, and forehand and backhand strokes were evaluated. The results demonstrated that both HIIT sessions achieved an average HR greater than 90% HRmax. The physiological demands (average HR) were greater during the playing session compared to the nonplaying session, despite similar lactate concentrations and a lower RPE. The results also indicate a reduction in shot velocity after both HIIT sessions; however, the playing HIIT session had a more deleterious effect on stroke accuracy. These findings suggest that 1) both HIIT sessions may be sufficient to develop maximal aerobic power, 2) playing HIIT sessions provide a greater physiological demand with a lower RPE, and 3) playing HIIT has a greater deleterious effect on stroke performance, and in particular on the accuracy component of the ground stroke performance, and should be incorporated appropriately into a periodization program in young male tennis players.

## Introduction

Tennis involves intermittent, high-intensity actions interspersed by periods of active and passive recovery [1]. High aerobic fitness is required to respond to the high demands of tennis players on the professional circuit [2]. High aerobic fitness is known to delay fatigue during repeated sprints [3], improving the rate of recovery between bouts of exercise, and is important in maintaining attention and concentration [4]. Therefore, the enhancement of VO_2_max is a crucial component of a tennis players conditioning program [5], and maybe implemented early in a tennis players career.

Sport specific interval training, characterized by distances and activities related to a tennis match may provide an appropriate stimulus to enhance VO_2_max [1]. As such, the inclusion of high intensity interval training (HIIT) based on game-specific on-court drills is thought to be ideal to enhance aerobic fitness while maintaining the technical skills, thus efficiently using training time [6,7]. This type of training is of interest for young tennis players, since the time devoted to conditioning is often limited [8], due to the extensive amount of time on the court practicing technical and tactical drills.

For a training session to effectively enhance VO_2_max, a physiological load between 80%-90% of VO_2_max, or 90%-95% of maximal heart rate [9] is required. Match play requires a mean oxygen uptake ranging from 46%-56% of VO_2_max, and a mean heart rate between 140–160 beats per minute [1]. Therefore, match play may not provide the required stimulus to enhance ones aerobic capacity. Game specific or playing HIIT training sessions that provide a higher physiological demand than those observed through match play are warranted. However, the fatigue associated effects on the technical skills during high intensity intermittent game specific conditioning sessions remains to establish. A pioneer study demonstrated that an 8-week playing HIIT training program improved VO_2_max, tolerance to fatigue and skilled tennis performance in adolescent tennis players [2]. Although these results are promising, the training protocol and the performance assessment presented two major limitations. First, the protocol did not account for the non-predictable trajectory of the ball because the players knew the location of the ball rebound [2]. Second, the tennis stroke performance assessment was only based on accuracy [2]. Tennis stroke performance assessment should evaluate both ball velocity as well as accuracy. As a consequence, the effects of a sport specific or playing HIIT session on shot performance, i.e. shot velocity and accuracy, must be determined before the inclusion in a conditioning program; especially for young tennis players who have limited training time and must efficiently train for both physiological and technical and tactical skills.

The purpose of this study was to compare the effects of playing and nonplaying high intensity intermittent training sessions of equal distance on physiological demands and tennis stroke performance in young competitive male tennis players. It was hypothesized that a physiological load required to enhance maximal oxygen uptake development will be reached by both playing and nonplaying HIIT. Secondly, due to the greater use of upper body musculature, it was hypothesized that the playing HIIT session would result in a greater deterioration in stroke performance compared to the nonplaying HIIT session.

## Materials and Methods

Eleven competitive male tennis players (age: 13.4 ± 1.3 years; height: 1.63 ± 0.10 m; body mass: 49 ± 10 kg; tennis experience: 7.8 ± 1.1 years; current weekly tennis training: 6.5 ± 1.0 hours; weekly conditioning training: 4.8 ± 1.1 hours; International Tennis Number: 3–4) volunteered. This study was approved by the French Ethics Committee Sud Est II. The players and guardians signed a written informed assent and consent, respectively. The baseline physiological characteristics of all the players were evaluated during the week preceding the beginning of the experimental procedure that lasted three weeks. During these three weeks, the players did not perform any training aimed at improving their VO_2_max. Resting heart rate (HR) was measured during a 10-min period while in a seated position using a HR monitor (RS 400, Polar, Kempele, Finland). Maximal HR (HRmax) and peak running velocity (Vmax_shuttle_) were determined at the completion of a 20-m shuttle run test [10]. Heart rate reserve (HRR) was subsequently calculated [11].

All players performed two HIIT sessions, in random order; a playing and a nonplaying HIIT session, separated by at least 48 hours. All exercise bouts were conducted on an indoor green set court, at the same time in the afternoon. Each session began with a 15-min standardized warm-up [12], followed by an evaluation of tennis stroke performance, as outlined below. After the stroke evaluation, each player performed either a playing or nonplaying HIIT session. Immediately following the session stroke performance was re-evaluated.

**Fig 1:**
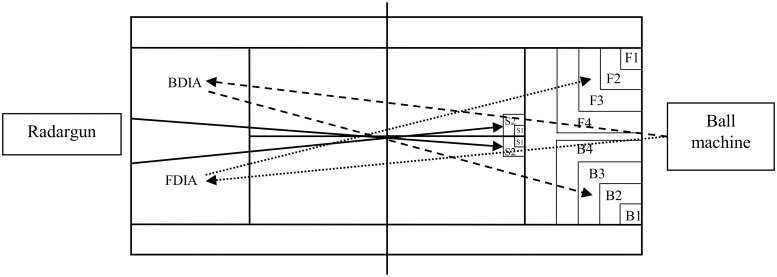
Tennis court instrumentation for stroke performance evaluation, with S1 and S2, the target areas for the serve; F1, F2, F3 and F4, the target areas for forehand drives; B1, B2, B3, B4, the target areas for backhand drives; FDIA, the forehand drive impact area, and BDIA, the backhand drive impact area. The full arrows indicate the ball trajectories for the serve, and the dotted arrows indicate the ball trajectories for forehand drive, and the dash arrows, the ball trajectories for backhand drive.

For the evaluation of stroke performance, the players performed one set of 12 serves (6 per diagonal), one set of 10 forehand strokes, and one set of 10 backhand strokes, performed in random order. They were instructed to hit first serves and groundstrokes as fast and as accurate as possible (one per type of stroke; Fig. 1), while focusing on achieving a winning shot. A radar gun (SR3600, Sports-radar, Homosassa, FL, USA) was located behind the players to record ball velocity. For the forehand and backhand stroke evaluations, tennis balls were projected by a machine (HighTOF, Echamboulains, France). To assess serve accuracy, a target was defined from the service and center lines, as previously described [13]. A ball bounce in the area S1 (0.5*0.5m) accounted for 5 points, S2 for 3 points; serve box for 1 point. Another location of the ball bounce resulted in zero point. To assess the accuracy of forehand and backhand strokes, the target was defined from the baseline and the alley line, as previously described [13]. A ball bounce in the area F1 or B1 (for forehand and backhand drives, respectively; 1*1 m) accounted for 5 points; F2 or B2 (2*2m) accounted for 4 points; F3 or B3 (3*3 m) accounted for 3 points; F4 or B4 (4*4m) accounted for 2 points; the opposite court accounted for 1 point. All other ball placements resulted in zero point. For each type of stroke, the accuracy was defined by the sum of all points, with a higher score corresponding to a higher accuracy. The stroke velocity corresponded to the mean velocity of all strokes.

The Vmax_shuttle_ were used to individualize the HIIT sessions. The HIIT sessions consisted of two 6-min sets interspersed with 5-min passive recovery. The first set was composed of a series of 10 s bouts at 110% of Vmax_shuttle_ and 20 s of passive recovery. The second set was composed of 15 s bouts at 105% of Vmax_shuttle_ and 20 s of passive recovery. The nonplaying HIIT session was conducted on the tennis court instrumented with four markers on the ground (see S1 Fig. for Tennis court instrumentation for HIIT sessions). The players began each set at the center of the baseline, running to each mark and touching it with one foot, and returning back to the baseline center before running to the next mark. The distances between the baseline center and each mark were individually calculated as a function of the individuals Vmax_shuttle_. For example, a subject with a 14 km∙h^-1^ Vmax_shuttle_ had to run 42 m at 110% of Vmax_shuttle_ during 10 s. For the playing HIIT session, instead of touching the marks with their foot, the players had to perform a tennis shot. The tennis balls were randomly projected by the ball machine in order that the players ran the same distance and performed the same number of directional changes during both HIIT sessions. Players were required to move as fast as possible, hit with maximal effort while maintaining stroke accuracy. During both sessions, the players were allowed to drink water ad libidum.

During both HIIT sessions, HR was recorded using a HR monitor (Polar RS800, Polar Electro, Finland), and analyzed with PolarPro Trainer 5 software (Polar Electro, Finland). Exercising HR data were expressed as percentage of HR reserve (%HRR). Because of the intermittent nature of the HIIT sessions, the mean HR alone may not be sufficient to characterize the aerobic demands. Therefore, the times spent in the 4 specified %HRR zones were recorded. HR was thus averaged by both 6-min sets, and the time spent in four zones of HRR during each set was computed: 80–85%, 85–90%, 90–95%, and 95–100%.

Blood lactate concentrations were measured 3-min after the warm-up, and 3-min after the second set, using a lactate analyzer (Lactate Pro, Arkay, Kyoto, Japan) [14]. Lastly ratings of perceived exertion [15], a subjective measure of intensity, was determined immediately after each HIIT session. The players were familiarized with the RPE scale one week prior to the experiment.

All data are presented as mean ± standard deviation (SD) (see S1 File for the raw data). After verifying normality, a repeated-measures analysis of variance (ANOVA) (HIIT session: playing vs. nonplaying; and time: pre- vs. post-test or 1st set vs. 2nd set) was applied to the physiological responses and tennis performance outcomes. When ANOVAs revealed a significant difference, p-value and partial eta square (η2), and their interpretation according to Cohen’s scale [16] (η2 = .01 for small effect, η2 = .06 for medium effect, and η2 = .14 for large effect) were reported. If a significant interaction was found, a Bonferroni post-hoc test was performed. A paired-t test was used to compare RPE between HIIT sessions. An effect size (ES) was computed and interpreted according to Cohen’s scale [16] (ES = 0.2 for small effect, ES = 0.5 for medium effect, and ES≥0.8 for large effect) for all pairwise comparisons. The level of significance was set at p ≤ 0.05. All analyses were performed on SPSS 11.0 (SPSS, Inc., Chicago, IL.).

## Results

### Heart rate responses

**Fig 2:**
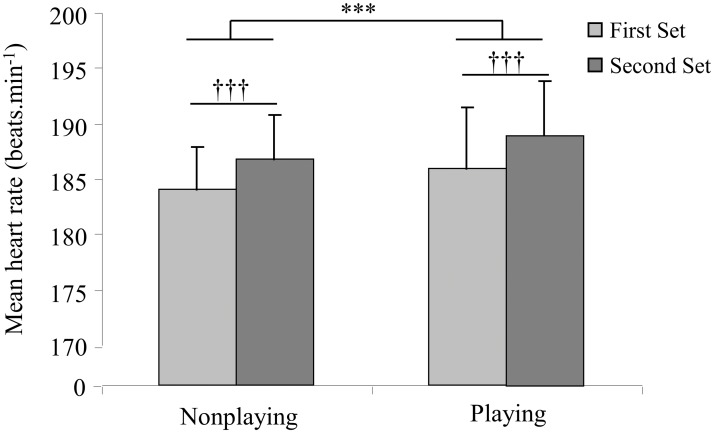
Mean heart rate (± standard deviation) for the first and second sets during the nonplaying and playing high intensity interval training sessions.

Resting HR was 70.7 ± 4.3 bpm. HRmax and Vmax_shuttle_ achieved at the end of the 20m shuttle run was 202.3 ± 3.1 bpm and 13.5 ± 0.9 km.h^-1^, respectively. Calculated HRR was 131.5 ± 6.5 bpm. For HIIT sessions, there was a significant large effect between HIIT sessions (p = 0.001; ² = 0.66, large effect) and between sets (p≤0.001; ² = 0.80, large effect) for mean HR; demonstrating a higher mean HR for the playing session when compared to the nonplaying session, and for the second 6-min set compared to the first set (Fig. 2). The mean percentages of HRmax were 91.6 ± 2.2% (ranged from 87.5% to 94.6%) for the nonplaying session and 92.7 ± 2.2% (ranged from 88.7% to 95.7%) for the playing session.

Time spent in each %HRR zones are shown in Table 1. There was a significant large effect of HIIT sessions and between sets for 80–85% (p = 0.006, η² = 0.69, large effect, and p = 0.03, η² = 0.48, large effect, respectively), for 85–90% (p = 0.03, η² = 0.47, large effect, and p = 0.05, η² = 0.41, large effect, respectively), and for 90–95% (p = 0.009, η² = 0.63, large effect, and p = 0.04, η² = 0.44, large effect respectively), while no significant effect was found for 95–100%. The time spent at 80–85% HRR was higher for the nonplaying session compared to the playing session, and for the second set in comparison with the first set. The time spent at 85–90% and 90–95% HRR was higher for the playing HIIT session and for the second set when compared to the first set.

**Table 1:** Mean (± standard deviation) time (s) spent in the four zones of heart rate reserve for the first and second sets of nonplaying and playing high intensity interval training sessions (n = 7).

	Nonplaying		Playing		
	1^st^ set	2^nd^ set	1^st^ set	2^nd^ set	
80–85%	103.6 ± 4.9	98.5 ± 6.0	96.4± 12.2	87.1 ± 17.0	**,†
85–90%	100.7± 5.3	105.6± 3.6	102.9± 4.8	108.5± 7.2	*,†
90–95%	70.7± 6.0	75.8 ± 7.2	76.5 ± 11.4	81.4± 11.5	**,†
95–100%	9.8± 3.0	10.1 ± 2.9	9.4 ± 1.9	12.2 ± 6.3	

* Training session effect with * for p≤0.05, and ** for p≤0.01.

† Set effect with † for p≤0.05.

### Blood lactate and Ratings of Perceived Exertion

The blood lactate concentrations were similar prior to the start of the training sessions (nonplaying: 3.8 ± 3.7 mmol∙L^-1^; playing: 3.6 ±3.2 mmol∙L^-1^), and both HIIT training sessions resulted in similar significantly large increases after the second set with 10.8 ± 3.6 mmol∙L^-1^ for the nonplaying session and 10.9 ± 3.3 mmol∙L^-1^ for the playing session (p≤0.001, η² = 0.99, large effect). After the completion of the HIIT session, RPE was 8.45 ± 0.7 for the nonplaying session, which was significantly higher than 7.67 ± 0.7 for the playing session (ES = 1.36, large effect; p≤0.001).

### Stroke Performance Evaluation

**Fig 3:**
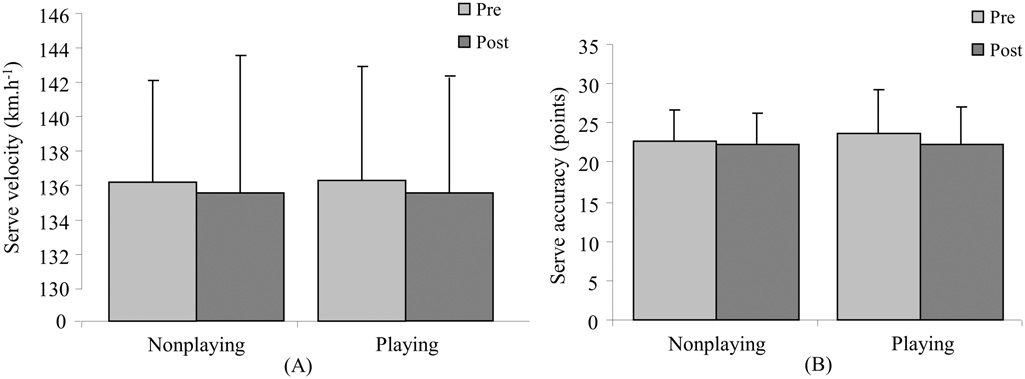
Mean (± SD) velocity (A) and accuracy (B) for serve tests before (pre-) and after (post) nonplaying and playing high intensity interval training sessions.

**Fig 4:**
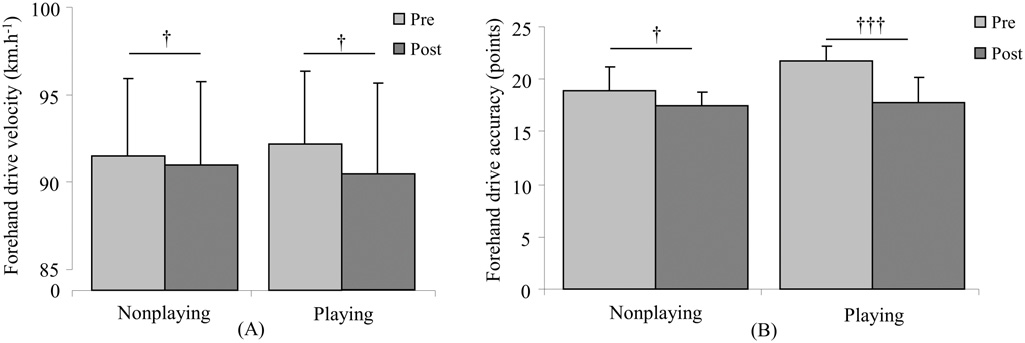
Mean (± SD) velocity (A) and accuracy (B) for forehand drive tests before (pre-) and after (post) nonplaying and playing high intensity interval training sessions. † represents time effect with † for p≤0.05 and ††† for p≤0.001.

**Fig 5:**
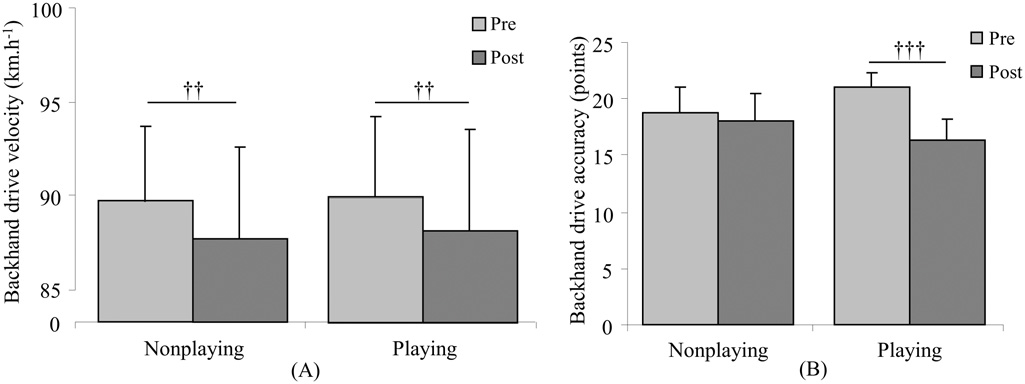
Mean (± SD) velocity (A) and accuracy (B) for backhand drive tests before (pre-) and after (post) nonplaying and playing high intensity interval training sessions. † represents time effect with †† for p≤0.01 and ††† for p≤0.001.

Concerning the effects of the HIIT sessions on stroke performance, there were no significant differences for either the velocity or accuracy of the tennis serve (Fig. 3); Significant time effect on both the forehand and backhand velocity (p = 0.02, η² = 0.35, large effect, and p = 0.004, η² = 0.58, large effect, respectively) was found. The interaction between HIIT sessions and time had significant effect on both forehand and backhand stroke accuracy (p = 0.02; η² = 0.39, large effect, and p = 0.001, η² = 0.72, large effect, respectively). For the groundstroke velocity (Figs. 4A and 5A), similar decreases were observed after the completion of both nonplaying and playing sessions (ES = 0.41, medium effect, and ES = 0.60, medium effect, respectively, for the forehand drive; ES = 1.00, large effect, and ES = 0.96, large effect, respectively, for the backhand drive), while the accuracy of groundstrokes (Figs. 4B and 5B) had a smaller decrease after the nonplaying HIIT session compared to the playing HIIT session (ES = 0.86, large effect, p = 0.02 and ES = 1.37, large effect, p = 0.001, respectively, for the forehand drive; ES = 0.30, small effect, p = 0.34 and ES = 2.65, large effect, p≤0.001, respectively, for the backhand drive).

## Discussion

The main results revealed that both on-court tennis specific HIIT sessions achieved the recommended HR required to enhance aerobic capacity. In addition, the mean HR and the time spent above 85% of HRR were greater during the playing HIIT session compared to the nonplaying session, despite a lower RPE. Concomitantly, the shot velocity decreased after both HIIT sessions, while the playing HIIT session had a more deleterious effect on stroke accuracy.

Since the aerobic fitness is an important requirement at a professional level [2], the development of VO_2_max may be scheduled early in the tennis player’s career. In this context, on-court playing interval training is believed to be a time efficient strategy to improve aerobic fitness while including both technical and tactical aspects of the tennis performance [1]. The present manuscript evaluated HIIT on court sessions designed to mimic the repetition of short rallies and periods of low intensity activity as observed during tennis match play, alternating 10 s—15 s running [17] at 110–105% of Vmax_shuttle_ followed by 20 s of passive recovery. The results of the present study found that the time spent at a high percentage of HRR (over 85%) was obtained after both HIIT protocols (Table 1); suggesting that HIIT provides sufficient stimulus to improve aerobic capacity, as previously observed in young competitive swimmers [18] and adolescent soccer players [19]. Comparing the literature examining HIIT training in tennis players is confounded by the utilization of various experimental and training designs (HIIT in the present study vs. repetition of 2-min bouts in Fernandez-Fernandez et al.’s study [6]). In addition, most of the studies in tennis physiology expressed the cardiac demand as a percentage of HRmax whereas we expressed it as percentage of HRR in order to limit the inter-individual variability [11]. The low cardiac demand (Fig. 2) at high HRR percentage (Table 1) in our study could be a result of the intermittent training protocol. It is well known that HR kinetics at the onset of exercise may be delayed; therefore, the high-intensity bouts of 10 s and 15 s utilized in the present study was likely not of sufficient duration to maintain HR at a very high level compared to a 2 min bout. However, the higher mean HR and times spent at high HRR during the second set, regardless the playing or nonplaying HIIT (Fig. 2) and controlling for running distance, suggest a HR drift. The present training sessions also required a large contribution through anaerobic metabolism, as noted from the high blood lactate concentrations (~11 mmol∙L^-1^), despite a less developed anaerobic system in young subjects.

By design, the total distance covered, the recovery time, and the number of directional changes were similar for both HIIT sessions. Consequently, the difference observed in HR between the two HIIT protocols (Fig. 2, Table 1) suggested that the higher cardiac demand for the playing HIIT session was most likely associated with the additional work of the forehand and backhand shots. This hypothesis is statistically strengthened by the large effect size across both HIIT sessions. The results of Botton et al. [20] reporting a significant contribution of the strokes in the total energy expenditure of tennis rallies also support this assumption. Bekraoui et al. [21] reported that running and striking the ball cost 10% more energy than running without striking the ball. Finally, the activation of trunk muscles [22] and potentially a restrained ventilatory pattern during forehand and backhand drives may also explain the higher HR during the playing HIIT. In addition, RPE was significantly lower after the playing HIIT session despite higher energetic demands. A reduced RPE at a similar physiological response was observed in small sided soccer games in comparison with in-line interval training [23]. This suggests that the tennis-specific features may decrease the perceptive difficulties of HIIT, and may help increase the motivation, as previously proposed [6], and strengthen the usefulness of playing HIIT sessions in young tennis players. In theory, the playing session may improve the adherence in specific training session in youth compared to general training session despite the similar physiological demand.

The present results also demonstrated that both HIIT sessions did not affect the tennis serve performance (Fig. 3). One explanation may be that neither session placed a specific demand on the primary muscles involved in the serving motion, such as subscapularis, pectoralis major, latissimus dorsi, and triceps brachii muscles [24,25]. In addition, the decrease in the groundstroke velocity after both HIIT sessions (Fig. 4A and 5A) may be related to the high cardiac demand that could alter the stroke biomechanics, in particular the timing of the cumulative power from legs to racket [26]. In contrast, the greater decrease in groundstroke accuracy was observed after the playing HIIT session (Fig. 4B and 5B) associated with the additional physiological loads on the upper limb generated by the ball hits. It could be then hypothesized that the repeated strokes during the playing HIIT session may progressively reduce forearm muscle activity [13] resulting in a decreased racket grip strength [27], and consequently a deficit in the control of the racket head at the time of impact. These latter results questioned the efficiency of such specific HIIT session for the improvement of aerobic fitness while maintaining the stroke performance in young tennis players. The conflict between the high cardiac demand and the decline in tennis ground stroke performance suggests that such specific training session may not concomitantly reach these two objectives. It may be however relevant to schedule such playing HIIT training sessions during the general conditioning period to highly solicit the aerobic system, while efficiently utilizing the training time and potentially improving the adherence of young tennis players to conditioning training.

This study presents several limitations that warrant discussion. The first limitation was associated with the sample population. Only young male tennis players competing at the national level were recruited for the present study. Therefore, the results may not be applied to the tennis players competing at other levels, female players or older players. We have to acknowledge that the maturity of the tennis players was not assessed in this study. Nevertheless, since the experimental design followed a test-retest procedure, each tennis player was his own reference for the pairwise comparisons, hence limiting the methodological bias in regards to the disparity in tennis player’s maturity. A second limitation concerned the test used to evaluate the incremental peak running speed. Indeed, the 20-m shuttle run test remains a general test, while the Hit & Turn Tennis Test [28] would be more specific. Furthermore, the stroke performance was not evaluated during the playing HIIT session. Although the players were instructed to hit the ball with maximal effort while maintaining the stroke accuracy during the playing HIIT session, inter-individual variability in stroke performance may induce an inter-individual variability in the cardiac response, as the stroke velocity can modulate energy expenditure [9]. Finally, despite the precautions to locate the radar on the tennis court, error of measurements could occur, in particular by the deviation between post-ball impact trajectory and radar directions. However, the current findings remain informative as this study was the first to investigate the effects of playing HIIT session on both physiological and stroke performance outcomes.

The primary purpose of playing HIIT is to combine improvement of physical conditioning and maintain the technical skills in order to optimize the training time, the findings of this study demonstrated that playing HIIT was able to sufficiently increase HR at a lower RPE; however, playing HIIT generated end-session decreases in tennis technical skills. Since the main goal of tennis-specific HIIT is to combine improvements of physical fitness and maintain technical skills in a time efficient manner; the results of the present study are important for tennis players and coaches whom should be aware that playing HIIT generated end-session decrements in tennis stroke performance. Therefore playing HIIT should be implemented appropriately into a periodization training plan. Further studies are warranted to examine the chronic effects on playing HIIT sessions and to evaluate strategies to reduce the decrement in stroke performance.

## Supporting Information

S1 Fig Tennis court instrumentation for HIIT sessions. (DOCX)

S1 File Raw data. (XLSX)
