# Supplementary material for: Playing vs. Nonplaying Aerobic Training in Tennis: Physiological and Performance Outcomes
Source: PLoS One. 2015 Mar 27;10(3):e0122718. doi: 10.1371/journal.pone.0122718 (PMC4376680; doi:10.1371/journal.pone.0122718)
Supplement: S1 Fig — (DOCX) [file pone.0122718.s001.docx]

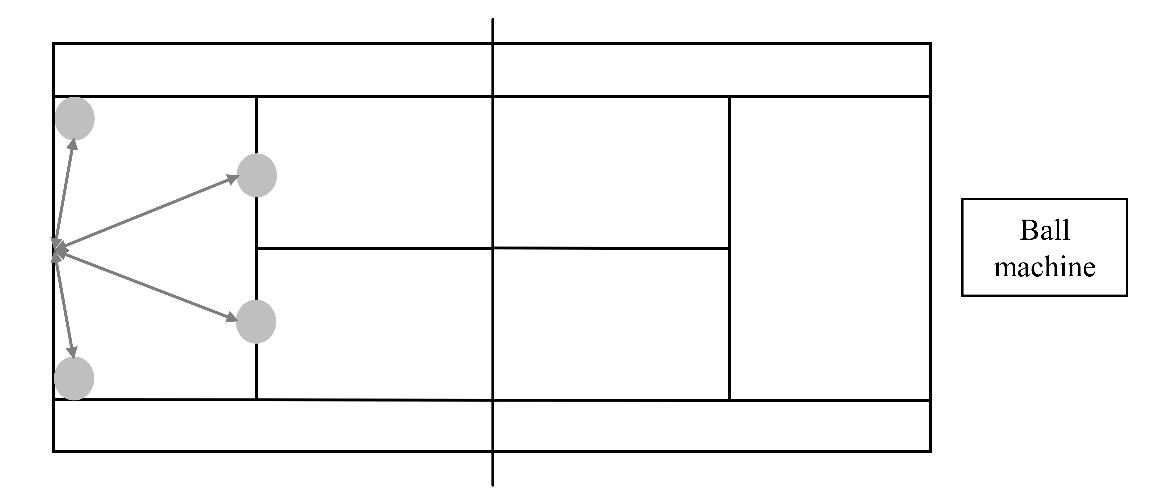


Figure S1: Tennis court instrumentation for HIIT sessions. Gray circles indicate the ground-marks for nonplaying HIIT sessions and impact areas for playing HIIT sessions. Arrows indicate the shuttle runs. The distances between the baseline center and ground-marks, and the number of shuttle runs were individually adjusted.
